# Multiple subcutaneous abscesses by *Corynebacterium amycolatum* in a patient with severe idiopathic aplastic anaemia

**DOI:** 10.1002/ski2.351

**Published:** 2024-02-14

**Authors:** Yuka Shintani, Ayano Fukushima‐Nomura, Takeru Funakoshi, Masatoshi Sakurai, Jun Kato, Masahiro Fukuyama, Toyoko Inazumi, Kiyofumi Ohkusu, Hayato Takahashi

**Affiliations:** ^1^ Department of Dermatology Keio University School of Medicine Tokyo Japan; ^2^ Division of Hematology Department of Medicine Keio University School of Medicine Tokyo Japan; ^3^ Department of Dermatology Tachikawa Hospital Federation of National Public Service Personnel Mutual Aid Associations Tokyo Japan; ^4^ Department of Microbiology Tokyo Medical University Tokyo Japan

## Abstract

*Corynebacterium amycolatum* is a part of the normal skin flora and has been underestimated as a pathogen. However, in recent years, the species has gained recognition as an important pathogen causing severe infections, particularly in immunocompromised patients. Nevertheless, identifying these organisms at the species level is difficult in routine clinical microbiology, leading to limited knowledge of their clinical manifestations in infectious diseases. In this study, we report a rare case of multiple subcutaneous abscesses in a patient with severe neutropenia, wherein *C.amycolatum* was identified as the causative organism through genotyping tests. This case highlights the importance of this organism as an aetiological agent of severe skin infections in patients with compromised immune systems.

## Graphical abstract

This manuscript presents a rare case report of a patient with severe idiopathic aplastic anaemia who developed multiple subcutaneous abscesses by *Corynebacterium amycolatum*. The report aims to elucidate the growing significance of *Corynebacterium* species as pivotal pathogens in individuals with compromised immune systems.

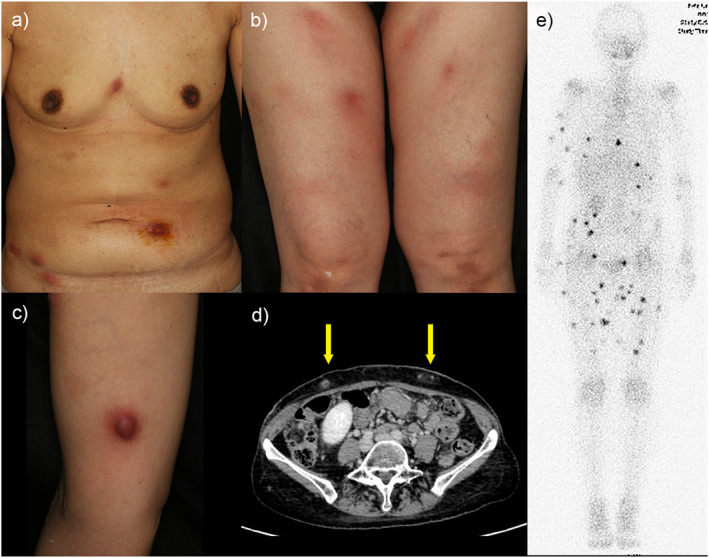

## 1 INTRODUCTION

*Corynebacterium* species are now recognised as essential pathogens in immunocompromised patients.^1^ In particular, *Corynebacterium amycolatum* has been increasingly reported to cause various infections and shows unpredictable multi‐drug resistance.^1^ This report presents a rare case of multiple subcutaneous abscesses due to *C. amycolatum* in a patient with severe idiopathic aplastic anaemia (IAA).

## 2 CASE REPORT

**FIGURE 1:**
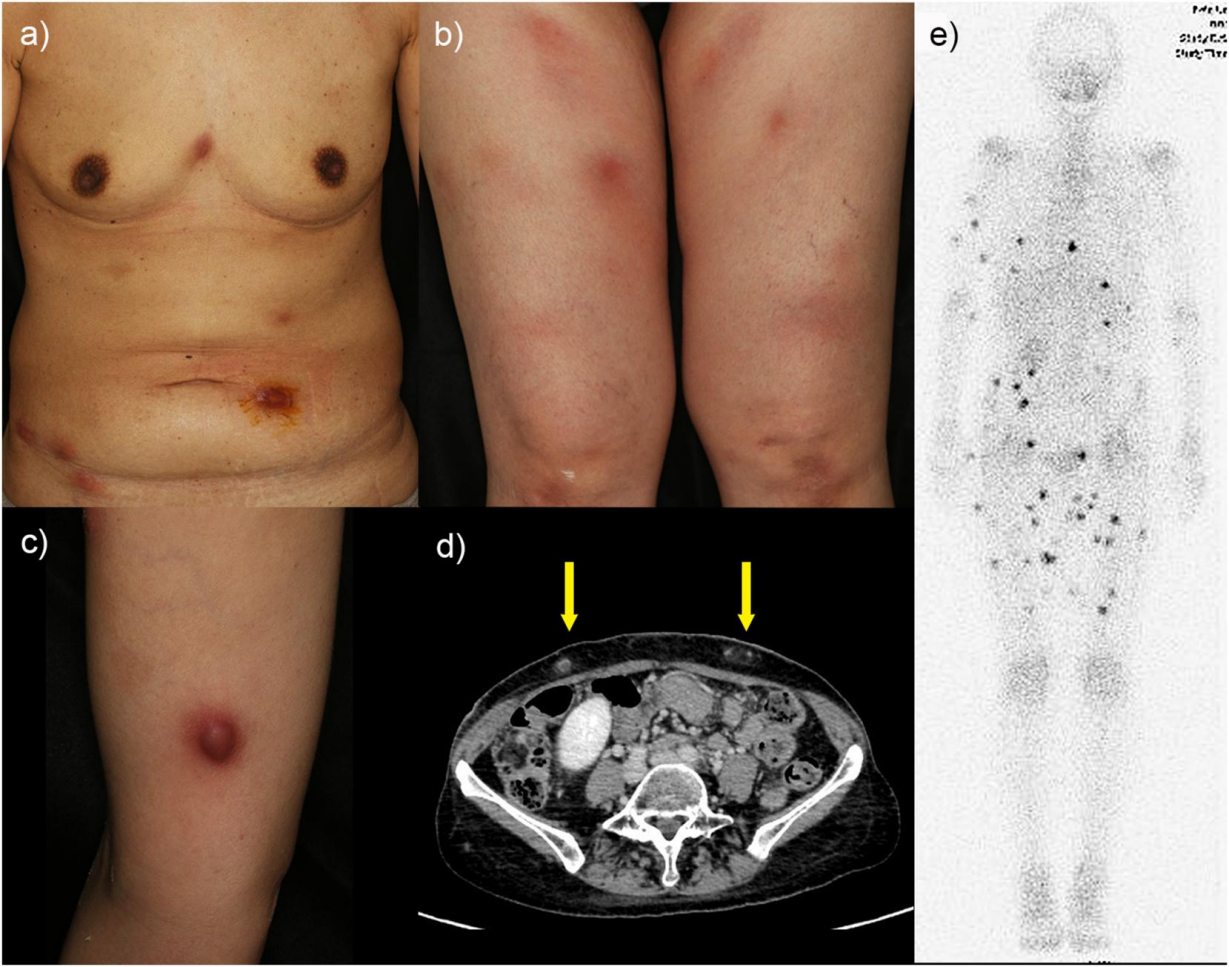
(a–c) Clinical images. Erythematous subcutaneous nodules are distributed on the whole body. The nodules were firm and tender. (d) The nodules (yellow arrow) were localised in the subcutaneous layer on computer tomographic scanning. (e) Gallium scintigraphy showed multiple nodular accumulations all over the body.

**FIGURE 2:**
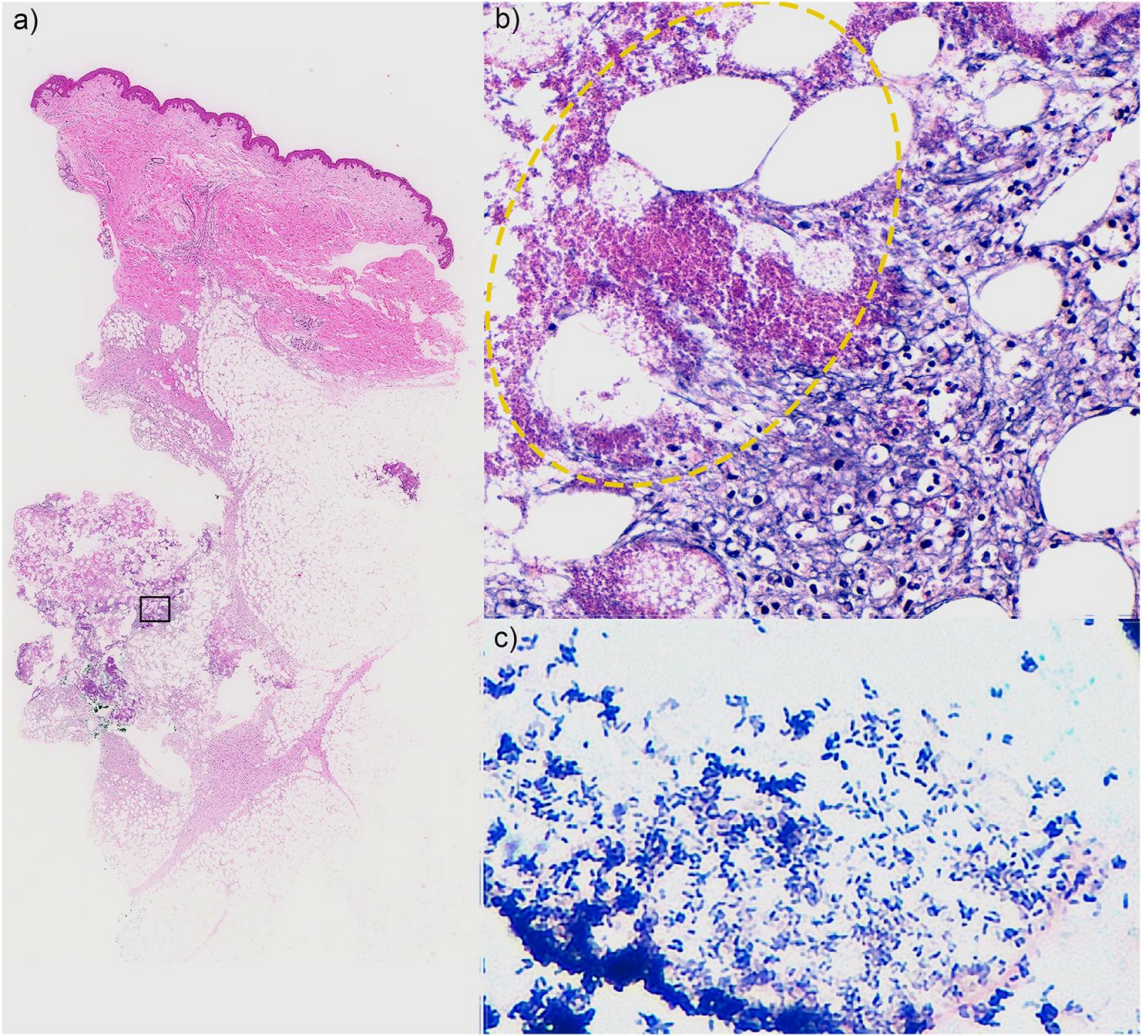
Histopathology of the skin biopsy. (a) Overview of the specimen. HE staining show cell infiltration and amorphous eosinophilic material deposition at the subcutaneous level. The region framed by the black box is magnified in (b). (b) High power view. The infiltration of lymphocytes accompanies the amorphous eosinophilic materials (shown in yellow circle). Neutrophils were hardly observed. (c) Gram staining showed gram‐positive rods in the areas of eosinophilic deposition seen in HE. Original magnification: (a) ×10; (b) ×100; (c) ×600. HE, Haematoxylin and Eosin.

A 51‐year‐old woman developed rapidly increasing multiple subcutaneous nodules on her trunk and extremities. She had been on immunosuppressant therapy for 2 months for severe IAA, with prophylactic treatment by levofloxacin. The nodules were up to 2 cm in size, erythematous, firm, and tender (Figure 1a–c). She presented with a fever of 38°C, severe neutropenia of 12/μL (normal range 1400–5950/μL), and almost normal C‐reactive protein of 0.9 mg/dL (0.3 mg/dL). The blood cultures were repeatedly negative. The computer tomographic scanning found no other organ involvement (Figure 1d). Gallium scintigraphy showed multiple nodular accumulations (Figure 1e). Upon skin biopsy incision, no pus was observed. However, the histopathology showed lymphocytic infiltration and amorphous eosinophilic material deposition in the subcutaneous level, which was identified as gram‐positive rods by gram staining, leading to the diagnosis of subcutaneous abscess (Figure 2). Although the tissue culture isolated ‘Coryneform bacteria’, API Coryne strip (bioMerieux, Marcy l’Etoile, France) testing could not identify the species. We additionally performed a complete 16S rRNA sequence analysis, revealing 99.2% identity with the type strain of *C. amycolatum*. The isolate showed resistance to levofloxacin, ampicillin, erythromycin, and clindamycin. Along with the recovery of peripheral neutrophils by immunosuppressive therapy for IAA, the nodules disappeared after 4 months of treatment: 1 month of intravenous antibiotics (meropenem and vancomycin, subsequently de‐escalated to ampicillin‐sulbactam) and 3 months of oral minomycin.

## 3 DISCUSSION

Skin and soft tissue infections in immunocompromised hosts pose a diagnostic challenge, as they develop atypical clinical manifestations under diminished immune response and can be caused by a wide array of microorganisms. Indeed, skin abscesses are caused not only by common pathogens such as *Staphylococcus aureus* but also by less common microbes such as Mycobacteria, fungi, and unusual bacteria such as *pseudomonas* and *Nocardia*.^2^, ^3^ Multiple lesions should be noted as they may indicate severe disseminated or systemic infection.^2^

Historically, *Corynebacterium* species have been underrated and ignored as a contaminant, as they are normal colonisers of the skin and generally show low virulence.^1^ *C. amycolatum* is also a part of the skin flora, which has recently been reported to manifest various infections such as sepsis, device‐associated infections, endocarditis, empyema, and breast abscess.^1^, ^4^ In skin and soft tissue infections, *C. amycolatum* has been isolated from wound and surgical site infections.^5^, ^6^ Cases in immunocompromised conditions can be fatal.^7^ In addition, *C. amycolatum* shows resistance to multiple antimicrobials, making treatments challenging.^1^ Clinical isolates are sometimes misidentified or unidentified by commercial phenotypic identification kits due to their variety of biological properties.^1^, ^8^ Accurate identification of the species warrants laboratory‐based genotypic tests, likely resulting in limited published cases reporting *C. amycolatum* as the culprit pathogen.^5^

To our knowledge, this is the first report of a *C. amycolatum* infection presenting with multiple subcutaneous abscesses. Although the blood culture was negative, considering the clinical features of multiple subcutaneous nodules spreading throughout the whole body, we inferred that bacteraemia preceded the skin manifestation. Our case raises awareness of the severe pathogenicity of *C. amycolatum* in skin infections of immunocompromised patients. Further reports are warranted in the understanding of infectious disorders caused by this species.

## CONFLICT OF INTEREST STATEMENT

None to declare.

## AUTHOR CONTRIBUTIONS

**Yuka Shintani**: Writing – original draft (lead). **Ayano Fukushima‐Nomura**: Writing – original draft (supporting). **Takeru Funakoshi**: Writing – review & editing (supporting). **Masatoshi Sakurai**: Writing – review & editing (supporting). **Jun Kato**: Writing – review & editing (supporting). **Masahiro Fukuyama**: Writing – review & editing (supporting). **Toyoko Inazumi**: Writing – review & editing (supporting). **Kiyofumi Ohkusu**: Writing – review & editing (supporting). **Hayato Takahashi**: Writing – review & editing (lead).

## ETHICS STATEMENT

Not applicable.

## Data Availability

Data sharing is not applicable to this article as no new data were created or analysed in this study.
